# Interplay Between the Cytoskeleton and DNA Damage Response in Cancer Progression

**DOI:** 10.3390/cancers17081378

**Published:** 2025-04-21

**Authors:** Clarissa Esmeralda Halim, Shuo Deng, Karen Carmelina Crasta, Celestial T. Yap

**Affiliations:** 1 Department of Physiology, Yong Loo Lin School of Medicine, National University of Singapore, Singapore 117593, Singapore; ce.halim@nus.edu.sg (C.E.H.); phsdes@nus.edu.sg (S.D.); karencrasta@nus.edu.sg (K.C.C.); 2 NUS Centre for Cancer Research (N2CR), Yong Loo Lin School of Medicine, National University of Singapore, Singapore 117599, Singapore; 3 Healthy Longevity Translational Research Programme, Yong Loo Lin School of Medicine, National University of Singapore, Singapore 117593, Singapore; 4 National University Cancer Institute, National University Health System, Singapore 119074, Singapore

**Keywords:** cytoskeleton, microfilaments, intermediate filaments, microtubules, DNA damage response, non-homologous end joining, homologous recombination, cancer, therapeutics

## Abstract

DNA damage has emerged as a critical factor in fuelling the development and progression of cancer. DNA damage response (DDR) pathways lie at the crux of cell fate decisions following DNA damage induction, which can either trigger the repair of detrimental DNA lesions to protect cancer cells or induce the cell death machinery to eliminate damaged cells. Cytoskeletal dynamics have a critical role to play and influence the proper function of DDR pathways. Microfilaments, intermediate filaments, microtubules, and their associated proteins are well involved in the DDR. For instance, they are not only implicated in the recruitment of specific DDR molecules to the sites of DNA damage but also in the regulation of the mobility of the damaged DNA to repair sites in the periphery of the nucleus. The exquisite roles that these cytoskeletal proteins play in different DDR pathways, such as non-homologous end joining (NHEJ), homologous recombination (HR), base excision repair (BER), and nucleotide excision repair (NER), in cancer cells are extensively discussed in this review. Many cancer treatments are reliant upon inducing DNA damage in cancer cells to eliminate them; thus, it is important to shed light on factors that could affect their efficacy. Although the cytoskeleton is intricately involved in the DDR process, this has often been overlooked in cancer research and has not been exploited in developing DDR-targeting cancer therapy. Understanding the interplay between the cytoskeleton and the DDR in cancer will then provide insights into improving the development of cancer therapies that can leverage the synergistic action of DDR inhibitors and cytoskeleton-targeting agents.

## Simple Summary

The cytoskeleton and its binding proteins help in the proper function of the main molecules of the DNA damage repair pathways. This promotes DNA damage repair in cancer cells that fosters their survival and cancer progression. This also prevents effective targeting of cancer cells with therapies dependent on the induction of DNA damage to cause their death. Therefore, understanding the roles that cytoskeletal molecules have in DNA damage repair pathways could stimulate the development of more effective cancer therapies by targeting both the DNA damage repair pathways and the cytoskeleton.

## 1. Introduction

Genome instability and the accumulation of mutations are key hallmarks of cancer that drive various oncogenic changes in cancer development [1]. While defective DNA repair machinery poses an important determinant in the accumulation of mutational load, external and internal stresses, such as oxidative stress, which could cause DNA damage, could also lead to a buildup of mutations. Unrepaired mutations in oncogenes and tumour suppressor genes could result in cellular transformation, with the gain-of-function of oncogenes and loss-of-function of tumour suppressors, thus driving carcinogenesis [2,3]. Once cancer has developed, the accumulation of DNA damage in the tumour cell could lead to two outcomes—the progression to a more aggressive disease due to increased genetic instability or the death of the cell due to extensive unrepairable damage [4,5,6]. One of the factors that decides this outcome is the DNA damage response (DDR) pathways [4]. Many cancer therapies rely on inducing extensive amounts of DNA damage, which exceeds the capacity of DDR pathways, to eliminate the tumour cells [7]. Possible DNA repair pathways that can be triggered depending on the type of DNA lesion include base excision repair (BER), mismatch repair (MMR), translesion DNA synthesis (TLS), nucleotide excision repair (NER), homologous recombination (HR), and non-homologous end joining (NHEJ) [8]. Cancer therapies that inhibit these DDR pathway processes and associated proteins, such as PCNA, 53BP1, and BRCA, among others, result in elevated DNA damage and cancer cell death. However, since DDR inhibitors target specific proteins, emerging tumour resistance ensues as the cells employ alternative DNA repair pathways to bypass the targeted mechanism [9]. The cytoskeleton has emerged as a critical player in many important cellular processes, and several studies have also exhibited that the cytoskeleton is involved in the regulation of the DDR. This provides an opportunity to explore the targeting of the cytoskeleton in conjunction with the DDR in cancer therapy as a means of overcoming the limitations of DDR inhibitors. Yet, the link between cytoskeletal dynamics and the DDR is not well focused on in cancer research. Therefore, in this review, we will highlight the ways by which various cytoskeletal molecules take part in the response to DNA damage. We will also discuss the potential for disrupting the cytoskeleton, and thus augmenting DNA damage or impairing the DDR, for an enhanced response to cancer therapy.

## 2. The Cytoskeleton and Cancer

The cytoskeleton provides essential structural and mechanical support to the cell, such as in regulating its shape and movement, organising its contents spatially, providing an anchorage and connection to its external environment, and aiding intracellular cargo transportation [10]. Apart from its biophysical roles, the cytoskeleton is also known to be involved in various biochemical processes of the cell and signalling pathways [11]. Since the cytoskeleton is not a static structure and the dynamic turnover of the monomeric and polymeric forms is crucial for the function of the cytoskeleton, the regulation of its dynamics is crucial in maintaining proper cellular processes and homeostasis [11]. Its dysregulation could lead to disease phenotypes such as cancer and its progression [12].

Microfilaments, microtubules, and intermediate filaments are the three major components of the cytoskeletal network. These three components, together with their associated proteins, form a mesh-like network of fibres within the cell, which is key to their function.

### 2.1. Microfilaments

Microfilaments, also known as actin filaments, are formed by polymerisation of either α, β, or γ-isoform actin molecules [13]. The monomer, globular actin (G-actin), nucleates and polymerises to form the filamentous form (F-actin), which is a 7 nm wide fibre [11]. F-actin is further organised into thicker bundles or networks of unbranched and branched structures by cross-linking proteins [11]. Since G-actin is always orientated the same way when it is being polymerised, F-actin has a polarity, and their ends are distinct from each other. It has a barbed (+) end on one side and a pointed (−) end on the other [13]. Actin polymerisation and depolymerisation occur on the barbed and the pointed end, respectively [13]. Under physiological conditions, the actin cytoskeleton does not stay as a static structure but rather undergoes constant remodelling and turnover. This modulation of actin dynamics, the nucleation, polymerisation, depolymerisation, and organisation, is carried out by numerous actin binding proteins (ABPs), such as the ADF/cofilin family, profilin family, and gelsolin superfamily of proteins as well as thymosins, DNase1, Arp2/3 complex, and other capping proteins [13,14]. Actin is the most abundant cytoskeletal protein in the cell and is essential for cell division by the formation of division rings, anchoring the cell to the extracellular matrix (ECM) with the aid of transmembrane proteins like integrins, cell motility by the formation of pseudopodia and lamellipodia, and vesicular transport within the cell with the help of its motor proteins, the myosins [14,15].

Actin and its ABPs are known to be implicated in the disease progression process in cancer [16,17]. Irregular actin remodelling during cell division, which results in F-actin accumulation preventing proper cytokinesis, could contribute to chromosomal instability leading to tumourigenicity [18]. Tumour metastasis also requires significant changes to the organisation of actin filaments to promote epithelial-mesenchymal transition (EMT) of the tumour cells for their migration and invasion to distant sites as well as mesenchymal-epithelial transition (MET) for the colonisation of the new sites [16]. Moreover, immune escape of tumour cells from targeting by cytotoxic T lymphocytes (CTL) and natural killer (NK) cells is also facilitated by the reorganisation of the actin network both in the tumour and immune cells [16]. Apart from these, changes in the expression level of the ABPs have been shown to help tumour cells inhibit apoptosis and promote cell proliferation, migration, invasion, and chemoresistance. For example, overexpression of gelsolin in various cancer cell types, such as human oral carcinoma, colorectal, cervical, gastric, and liver cancers, increased cell proliferation and invasion and suppressed their apoptosis [19,20,21,22,23,24]. Gelsolin could also regulate the susceptibility of human head and neck cancer to cisplatin treatment [25]. Cofilin could promote cell migration in different tumour types [26,27]. The RhoGTPase Rac likewise increased tumour cell migration through actin polymerisation dependent on the Arp2/3 complex [28,29].

### 2.2. Microtubules

Microtubules are hollow, tubular structures with a diameter of 25 nm formed by the polymerisation of α- and β-tubulin heterodimers [11,30]. α- and β-tubulin monomers heterodimerise and subsequently polymerise into a linear protofilament, which associates with adjacent protofilaments laterally to form the tubular structure [31]. In humans, there are seven isotypes of α-tubulin and eight isotypes of β-tubulin, whose expression is cell-type specific [32]. Unlike its α- and β-tubulin counterparts, the two isoforms of γ-tubulin do not participate in the polymerisation into the tubules. The γ-tubulin localises at the microtubule organising centre (MTOC) in the centrosome and is necessary in the nucleation process for the assembly of the microtubules [32,33,34]. Similar to actin, microtubules have a polarity with a fast-growing plus (+) end and a slower-growing minus (−) end [34]. They also undergo dynamic polymerisation and depolymerisation continuously. The functions of microtubules are similar to the other major cytoskeletal families of proteins: they are important in providing mechanical support to the cell, cell movement, formation of cilia and flagella, intracellular trafficking of cargo, and formation of the mitotic spindle during cell division [30,33,34]. However, unlike microfilaments and intermediate filaments, microtubules are not organised in vast networks throughout the cell [34]. Individual microtubule functions in a small cluster or on its own [34]. Microtubule binding proteins (MTBPs) modulate the dynamic assembly and disassembly of microtubules, contributing to their inherent dynamic instability. They consist of stabilisers (microtubule-associated proteins (MAPs), e.g., MAP1, MAP2, tau family of proteins), destabilisers (katanin, stathmin, kinesin 8 and 13), capping proteins, bundlers and cross-linkers (MAP65), and cytoskeletal integrators that bind to the other cytoskeletons (APC, plakin family of proteins, tau) [31].

Changes in the expression level of tubulin isoforms as well as in their post-translational modifications (PTMs) in cancer have been shown to be implicated in the progression of the disease [35]. In breast cancer, increased expression of βI-tubulin led to increased resistance to treatment with docetaxel, and detyrosinated tubulin is associated with a more aggressive disease [36,37,38]. Dharmapal et al. discovered that a βIIC-tubulin isotype helps to sustain the cancer stem cell (CSC) population in oral carcinoma [39]. High βIII-tubulin expression has been linked to increased chemoresistance, poor survival, and advanced cancer stage in non-small cell lung cancer (NSCLC), ovarian, prostate, and gastric cancer [40,41,42,43,44,45,46]. Increased acetylation of α-tubulin also resulted in increased resistance to paclitaxel in lung cancer [47]. Alteration to the expression level of MTBPs could also lead to cancer progression. Breast cancer cells that have a high tau expression are more resistant to paclitaxel treatment [48]. Low MAP2 levels are also correlated to resistance towards microtubule-targeting drugs in cancer cells, for example, in melanoma and pancreatic cancer [49].

### 2.3. Intermediate Filaments

The intermediate filaments (IFs) are made up of six classes of tissue-specific proteins [50,51]. Acidic and basic keratins in epithelial cells belong to Class I/II of IFs [51,52]. Class III includes vimentin in mesenchymal cells, desmin in muscle cells, glial fibrillary acidic protein (GFAP) in glial cells and astrocytes, among others. Class IV is composed of neurofilaments in the central nervous system, while nuclear lamins and nestin in neuroepithelial cells are in Class V and VI of IFs, respectively [52,53]. The intermediate filaments have a diameter of 10 nm and are formed by the assembly of four protofibrils, which are in turn made up of the organisation of two protofilaments together [51]. The protofilaments are assembled from tetramers of fibrous IF polypeptides that have homo- or heterodimerised [52]. IFs, unlike microfilaments and microtubules, do not have plus or minus ends and are thus apolar [52]. They have a more stable filamentous structure and act as a scaffold to both actin and microtubules [50]. This gives mechanical structure to support the cells. IFs are resistant to mechanical forces and help the cells to withstand these stresses [50]. They also play a role in cell-cell contact through desmosomes and cell-ECM junctions through hemidesmosomes [50]. Unlike microfilaments, intermediate filaments do not play a direct role in regulating cell movement.

In cancer, vimentin is widely acknowledged as the intermediate filament that is a mesenchymal marker promoting tumour cell migration, invasion, and metastasis in multiple cancer types [54]. It has been implicated in the progression of breast, lung, prostate, bladder, and gastrointestinal cancers and melanoma [54,55,56,57]. The overexpression of vimentin in these cancer cells promotes their EMT, leading to metastasis and poor prognosis [55,56]. Vimentin has been found to drive tumour cell invasion by cross-linking to the F-actin forming the invadopodia through plectin, thus stabilising their formation [56]. Apart from vimentin, nestin has also been implicated in the disease progression of several cancer types, for example, breast, colorectal, uterine, gastrointestinal, and pancreatic cancers. Nestin is expressed in CSCs and can be used as a biomarker to detect them [58]. It also promotes cell proliferation, survival, angiogenesis, and invasion of CSCs [59,60]. Keratins have long been used as a marker for epithelial tumour cells since they maintain the expression of keratins specific to their original cell type [61]. Moreover, several types of keratins have been found to impact the progression of cancers. Keratin K17 could translocate to the nucleus, and its high expression confers increased drug resistance in tumours, leading to poor survival [62]. K16 also promotes the EMT of breast cancer cells and thus is involved in their metastatic progression [63].

## 3. DNA Damage Response and Cancer

Deficiencies in the DNA Damage Response (DDR) can confer genomic instability that predisposes cells to acquiring tumourigenic mutations [6]. However, these deficiencies also have an effect on the response of cancer cells to chemotherapeutic treatments and, in some cases, have been linked to the regulation of their metastatic progression [5,6]. Thus, not only is the DDR important in the cancer initiation stage, but it also plays a critical role in cancer progression. The major DDR pathways in humans include base excision repair (BER), mismatch repair (MMR), translesion DNA synthesis (TLS), nucleotide excision repair (NER), homologous recombination (HR), and non-homologous end joining (NHEJ), as summarised in Table 1 [8].

BER is the pathway responsible for the repair of DNA single-strand breaks (SSBs), which are caused by reactive oxygen species (ROS), alkylation, depurination, or deamination [64]. The damaged base is first excised by a DNA glycosylase, leaving behind an abasic site for APE1 to cleave [64]. DNA polymerase β and XRCC1 then fill in the excised site [64]. BER has been found to be impaired in prostate and breast cancer due to defects in a key glycosylase, oxoguanine glycosylase 1 (OGG1) [65,66,67]. In mice, double knockout of glycosylases OGG and MUTYH causes the mice to develop lymphomas and liver and ovarian tumours [68].

DNA replication mistakes resulting in the wrong pairing of bases are repaired by MMR [6,69]. The clamp protein, proliferating cell nuclear antigen (PCNA), helps MSH2-MSH6 or MSH2-MSH3 heterodimers bind to the mismatched site, initiating repair [69,70]. Exo1 and DNA polymerase δ subsequently recruit the MLH1-PMS2 heterodimer to excise the error and resynthesise DNA with the correct bases [69,70]. Defective MMR due to mutations in its key players like MSH2, MLH1, and MSH6 are found in hereditary non-polyposis colorectal cancer (HPNCC) and endometrial cancer, while the epigenetic silencing of MLH1 could also cause sporadic colon cancers [71,72,73,74].

NER helps to repair exogenous DNA damage caused by ultraviolet (UV) light and bulky adducts due to chemicals. There are two NER types—global genome NER (GG-NER) and transcription-coupled NER (TC-NER) [75]. DNA helix distortions, recognised by XPC and RAD23B, initiate GG-NER, while the stopping of RNA polymerase II due to a lesion will initiate TC-NER [75,76]. Following this, the transcription factor IIH (TFIIH) complex comprising of XPA, XPB, and XPD is recruited to the lesion, while DNA-binding replication protein A (RPA) binds to the undamaged strand. XPF-ERCC1 dimer cleaves the DNA 5′ to the lesion, while XPG cleaves it on the 3′ end, thus removing the lesion [75]. This gap will be filled by DNA polymerases. Defects in this repair pathway lead to *Xeroderma Pigmentosum* and predispose the individuals to a high risk of skin and other cancers [75].

In TLS, a number of specialised DNA polymerases, such as DNA polymerases η, κ, ζ, θ, λ, and Rev 1, are recruited to bypass a lesion so that replication can continue without first having to repair the damage [77,78,79]. This occurs in the presence of DNA interstrand cross-links (ICL) [80]. In different phases of the cell cycle, the initial response to ICL involves the engagement of other DDR pathways, NER in G1 or HR in S/G2 phase, before TLS polymerases are recruited by PCNA [80,81]. The presence of ICLs predisposes an individual to increased cancer risks, e.g., acute myeloid leukaemia (AML). Resolving ICLs will also prevent the cells from undergoing mitotic catastrophe or apoptosis [80]. However, cancer cells have been found to rely on TLS polymerases for DNA replication during proliferation [6,82]. Many of them are overexpressed in various cancer types, which increase drug resistance and are associated with poor outcomes in patients [83,84,85,86,87,88,89,90,91,92].

Unrepaired DNA damage can result in double-stranded breaks (DSBs), the most cytotoxic DNA lesion [93]. DSBs are repaired by HR and NHEJ pathways. NHEJ is more commonly used by the cell than HR, especially in the G0/G1 phase of the cell cycle due to the difficulty in getting the homologous chromosomes in close proximity to each other for HR to take place [93,94]. In comparison to NHEJ, HR allows for a more accurate repair and is preferred to NHEJ at DNA replication forks [94,95]. The choice between NHEJ and HR is mediated by Ataxia Telangiectasia Mutated (ATM) signalling. Dependent on the structure of the DNA at the DSB sites, the MRE11/RAD50/NBS1 (MRN) complex binds to the DSB and recruits ATM to phosphorylate respective downstream targets to activate either NHEJ or HR pathways [73,74]. ATM’s phosphorylation of γH2A.X and 53BP1 inhibits HR and favours the engagement of NHEJ [95]. On the other hand, BRCA1 phosphorylation by ATM promotes HR repair and inhibits NHEJ [95,96].

NHEJ involves Ku70 and Ku80 heterodimers binding to the ends of the DNA at the DSB. The heterodimer recruits the catalytic DNA-PKcs and XRCC4-DNA Ligase IV complex to carry out gap-filling and ligation of the break, thus repairing the DSB [93]. In cervical cancer, it was found that the increased expression of NHEJ proteins confers the tumour cells with more resistance against irradiation [97]. Tumour cells also use NHEJ to promote their survivability, and its inhibition prevents cancer progression [98]. Suppression of the NHEJ repair pathway sensitises tumour cells to chemotherapeutic drugs [99].

HR requires a homologous template, in the form of either sister chromatids or homologous chromosomes to be proximally present for the repair to occur [94]. On the damaged chromosome, the MRN complex generates 3′ overhangs at the DSB site, which would facilitate DNA strand pairing with the homologous template [93]. BRCA1 subsequently recruits BRCA2 to load RAD51 to the DSB [94]. RAD51, along with RAD52, RAD54, WRN, and BLM, coordinate the pairing of the homologous strands of DNA for the repair process to take place [94]. It is well known that mutations in BRCA1 and BRCA2 predispose individuals to a higher risk of early-onset breast and ovarian cancers [5]. A number of mutations in different key players of HR also confer a higher predisposition to various cancers [100].

Apart from these DDR repair pathways, another response of the cell to DNA damage is to activate its cell cycle checkpoint pathway. Cell cycle arrest allows the cell ample time to repair the DNA damage or induce its apoptosis when repair fails so that the errors are not passed down to daughter cells [95]. The cell cycle checkpoint pathway is also activated by ATM when DSBs are present [6,95]. ATM activates Chk2, p53, and p21 to induce cell cycle arrest [101]. The diverse substrates of ATM prove the importance of its function within the DDR pathway and are a critical factor that mitigates multiple DNA damages. In totality, ATM functions to coordinate the cell’s response to DNA damage, including the determination of repair pathway choice, cell cycle checkpoint activation, apoptosis, and senescence, among others [95,96,102]. Hence, in cancer cells, ATM is often upregulated [103]. The increase in ATM signalling promotes cancer cell survival, metastasis, and resistance to cancer therapies [103,104,105,106,107].

**Table 1:** DDR pathways and their key molecules.

DDR Pathway	DNA Damage Type	Key Players
BER	SSB due to ROS, alkylation, depurination, deamination	DNA glycosylases, APE1, DNA Pol β, XRCC1 [64]
MMR	Wrong base pairing due to DNA replication mistakes	PCNA, MSH2, MSH3, MSH6, MLH1, PMS2, Exo1, DNA Pol δ [69,70]
TLS	Interstrand cross-links	PCNA, DNA Pol η, κ, ζ, θ, λ, Rev1 [77,78,79]
NER	DNA damage caused by UV radiation and bulky adducts due to chemicals	RAD23B, XPA, XPB, XPC, XPD, XPF, XPG, TFIIH complex, RPA, ERCC1 [75,76]
HR	DSB during S/G2 phases of the cell cycle	BRCA1, BRCA2, RAD51, RAD52, RAD54, WRN, BLM [94]
NHEJ	DSB during G0/G1 phases of the cell cycle	γH2A.X, 53BP1, Ku70/80, DNA-PKc, XRCC4, DNA Ligase IV [93,95]

## 4. The Roles of the Cytoskeleton in DDR

### 4.1. Microfilaments and DDR

**Figure 1:**
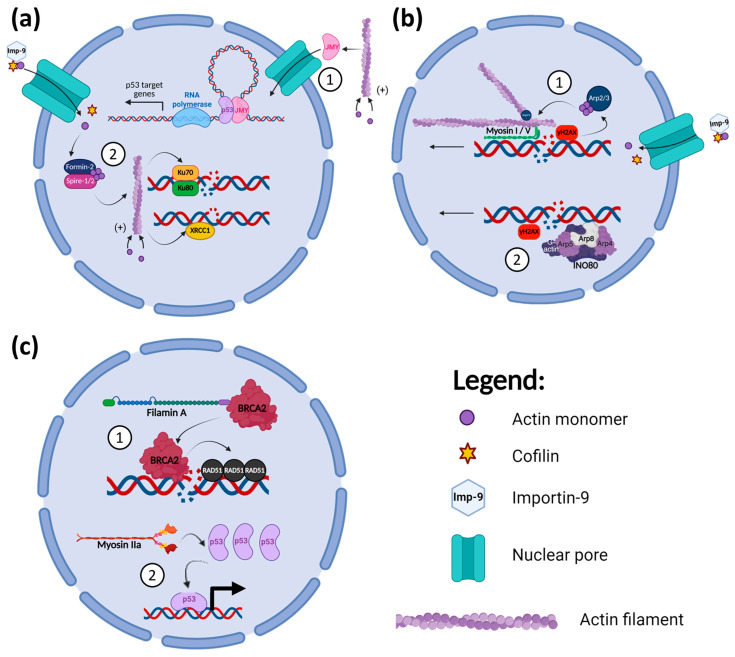
Microfilament roles in the DDR. (**a**) Actin polymerisation—1. Actin polymerisation promotes JMY nuclear translocation to augment p53 target gene expression. 2. Nuclear actin translocation and polymerisation by Formin-2 and Spire-1/2 increase the recruitment of DDR proteins, Ku70/80 and XRCC1, to sites of DNA damage. *Created in BioRender. H, C.E. (2025)* https://BioRender.com/8q6h0sy (accessed on 14 April 2025). (**b**) Microfilament proteins aid in the movement of damaged DNA to the nuclear periphery for repair—1. Arp2/3 nucleates actin filaments close to the DSB sites, where myosin I/V helps in the sequestering of the DSB to the nuclear periphery. 2. Actin and its ABPs are subunits of the chromatin remodeller complex, INO80, which facilitates the translocation of the damaged DNA. *Created in BioRender. H, C.E. (2025)* https://BioRender.com/6fytb6a (accessed on 14 April 2025). (**c**) Roles of ABPs in the DDR—1. Filamin A interacts with BRCA2, promoting its recruitment to DSB sites targeted for HR, which increases RAD51 recruitment. 2. Myosin IIa regulates the retention of p53 in the nucleus in the presence of DNA damage, promoting the expression of p53 target genes. *Created in BioRender. H, C.E. (2025)* https://BioRender.com/j52h155 (accessed on 14 April 2025).

The actin cytoskeleton not only forms various structures in the cytoplasm but is also present in the nucleus. Actin constantly shuffles between the cytoplasm and nucleus to regulate its concentration in both compartments [108]. This is mediated by Importin-9 with cofilin for the entry of actin into the nucleus and Exportin-6 with profilin for its exit [108]. Nuclear actin and ABPs have been discovered to play multiple roles in the DDR, as illustrated in Figure 1. Their functions could be classified broadly into three areas: (1) recruitment of DDR proteins, (2) sequestering of damaged DNA sites towards the nuclear periphery for HR repair, and (3) subunits of chromatin remodeller complex that modulate DSBs mobility.

In response to DNA damage, actin polymerisation increases in both the cytoplasm and nucleus [109,110]. Cytoplasmic actin polymerisation promotes the translocation of junction-mediating and regulatory protein (JMY), a multifunctional actin regulator, into the nucleus in response to DNA damage, as shown in Figure 1(a1) [109]. JMY contains three actin-monomer binding domains, the WH2, which are essential for nucleating actin polymerisation [109]. The polymerisation of actin depletes its monomers in the cytoplasm, freeing JMY’s WH2 domain to bind to importin and permitting its movement into the nucleus [109]. Within the nucleus, JMY functions as a transcriptional co-activator of p53, a downstream target of the ATM pathway, increasing the expression of p53 target genes, such as *XPC*, *XRCC5*, and *TP53I3* [111,112]. The increase in p53 target gene expression by JMY augments the efficiency of DNA repair, which allows for continued cell proliferation and the prevention of cell death [112]. Hence, cancer patients with higher JMY expression in tumours have a lower overall survival [112].

On the other hand, actin polymerisation in the nucleus induced by DNA damage helps in the efficient repairing of DSBs, as illustrated in Figure 1(a2) [110]. Nuclear actin filaments are maintained by the nuclear translocation of cytoplasmic actin monomers and their nucleation by Formin-2 or Spire-1/2 in the nucleus [110]. Ku70/80 interacts with the nuclear actin filaments to promote NHEJ repair [113]. The disruption of actin dynamics by cytochalasin D shortened the retention of Ku80 at DSBs [113]. Additionally, Hurst et al. (2021) showed that the inhibition of nuclear actin polymerisation reduced the accumulation of XRCC1 to sites of DNA damage, thus preventing efficient repair through BER [114]. In colorectal cancer cells treated with Zeocin, a DNA-nicking and base-altering drug, cell proliferation is decreased when actin polymerisation is impaired [114]. Therefore, nuclear actin filaments aid in both DSB repair and BER of the DDR, promoting the cell proliferation of cancer cells even in the presence of DNA damage.

Actin and its nucleation factor Arp2/3 are also needed for efficient HR repair of DSBs [115]. DSBs directed for HR repair in the G2 phase of the cell cycle recruit Arp2/3 complexes to mediate the nucleation of a highly branched actin network near the DSBs [115]. γH2A.X-mediated signalling then recruits nuclear myosin I and V to bind to the damaged DNA [116,117,118]. This modulates the movement of the DSBs along the actin filament to the nuclear periphery, allowing them to cluster together for efficient DSB end resection and HR repair to take place [115,119]. The nucleation of actin by Arp2/3 to promote HR is independent of the Formin-2 and Spire-1/2 activities discussed above [115]. The inhibition of myosin I’s motor function prevents its binding to damaged chromatin, thus blocking the relocation of DSBs along actin filaments within the nucleus [116,117]. These studies show that DSBs movement mediated by actin filaments and their myosin motors are necessary for their efficient repair through HR, as shown in Figure 1(b1). Preventing DSBs clustering by the inhibition of Arp2/3 also sensitises cancer cells to various DNA damaging agents [115], highlighting the crucial roles of nuclear actin in DNA damage management by cancer cells.

Another mechanism by which actin regulates HR is via its interaction with the INO80 remodeller, as illustrated in Figure 1(b2). The INO80 complex chromatin remodeller is made up of a number of protein subunits, including actin and its ABPs, Arp4, Arp5, and Arp8 [120,121]. The INO80 complex remodels the chromatin upon DNA damage, increasing the mobility of nucleosomes, thus promoting the movement and translocation of DNA with DSBs to the nuclear periphery for HR repair [108,120]. In the DNA damage response, γH2A.X recruits the INO80 complex to bind to the chromatin proximal to the DSB sites [122]. Arp8 is the key DNA-binding subunit in the complex, which binds to both double- and single-stranded DNA (dsDNA and ssDNA), with a preference for the latter [122,123]. Arp4, Arp5, and Arp8 also bind to histones, and their presence in the INO80 complex is necessary for its chromatin remodelling activity [121]. The monomeric actin subunit in the complex is also essential for its chromatin remodelling function [124]. A mutation in the actin subdomain 2 reduced the capability of the INO80 complex to bind to free DNA and nucleosomes, as well as attenuating its ATPase activity [124].

In addition to its role in repairing DSBs, the INO80 complex is also involved in the NER pathway [125]. The INO80 complex is recruited to UV lesions and aids in the subsequent recruitment of XPA and XPC of the NER pathway [125]. The Arp5 subunit of the complex is necessary for the proper engagement of NER to repair UV irradiation damage of the DNA [125]. The INO80 complex has also recently been elucidated to enhance the activity of the AP-endonuclease 1 of the BER pathway [126]. However, it is not known if the actin and Arps subunits play key roles in this function of the complex.

Other ABPs that have been discovered to be associated with the DDR process are Filamin-A, an actin cross-linking protein, and myosin IIa (Figure 1c). Filamin-A has a BRCA2 interacting domain on its C-terminus, and this interaction modulates the efficiency of HR repair of DSBs [127,128]. Filamin-A null melanoma cells are more susceptible to radiation than Filamin-A proficient cells [128]. They have increased and more persistent γH2A.X foci with more chromosomal aberrations due to a decreased formation of RAD51 foci after irradiation [128]. Filamin-A re-expression in the Filamin-A null melanoma cells increased HR RAD51 foci formation and cell survival after γ-radiation [128]. Moreover, Filamin-A knockdown in other melanoma and breast cancer cells decreased their survivability post-DNA damage induction. Thus, Filamin-A can contribute to the resistance of tumour cells to γ-irradiation [128]. Schramek et al. (2014) reported that non-muscle myosin IIa is involved in the DDR by regulating p53 retention and accumulation in the nucleus following DNA damage, independent of actin dynamics disruption by latrunculin B [129]. A deficiency of myosin IIa or its inhibition by blebbistatin prevented the accumulation of p53 in the nucleus. This blocks the transcription of p53 target genes regulating the DDR cell cycle checkpoint pathway, such as p21 [129]. In head and neck squamous cell carcinoma (HNSCC), the expression of the non-muscle myosin IIa heavy chain gene is often downregulated, which is associated with lower survival [129]. The loss of myosin IIa expression also promotes increased metastasis of SCC [129].

Several studies have also implicated Anillin, another ABP that usually plays a role in cytokinesis, in the DDR. Anillin has been found to interact with BRCA1 [130]. Its downregulation in gastric cancer cells has also been linked to the inhibition of DDR pathways, leading to an accumulation of irreparable DNA damage in the S-phase of the cell cycle, thereby promoting cancer cell death [131]. Similarly, the knockdown of Anillin in hepatocellular cancer cells prevented cytokinesis and increased DNA damage, resulting in apoptotic cell death [132]. Another report also demonstrated that p53 induces the repression of Anillin in response to DNA damage [133]. One could thus envisage the use of Anillin inhibitors to promote tumour cell apoptosis for cancer therapy. However, a more detailed study is required to elucidate the exact function of Anillin in the DDR pathways and how its expression levels affect the response of cancer cells to DNA damage.

### 4.2. Microtubules and DDR

Several studies have also discovered an interplay between the DDR and microtubule dynamics. A number of DDR proteins, such as DNA-PK, ATM, Chk1/2, and BRCA1, are localised to the centrosome during mitosis [134]. DNA-PK, in particular, is located in the centrosome even during the interphase stage of the cell cycle [134]. BRCA1 interacts with γ-tubulin both at the centrosome as well as intranuclear sites [134]. These DDR proteins are found to regulate the nucleation and polymerisation of microtubules. A study by Ma et al. (2021) displayed that 53BP1 and DNA-PKs are required to mediate microtubule polymerisation in response to DNA DSBs in the G0 and G1 phases of the cell cycle [135]. This regulation of microtubule dynamics by DDR proteins is dependent on DNA-PK phosphorylating and activating Akt in the centrosomes to induce the nucleation and polymerisation of microtubules [135]. The inhibition of DNA-PK, on the other hand, prevents the efficient recovery of microtubule dynamics after treatment with nocodazole and instead prompts the translocation of α-tubulin into the nucleus [134].

**Figure 2:**
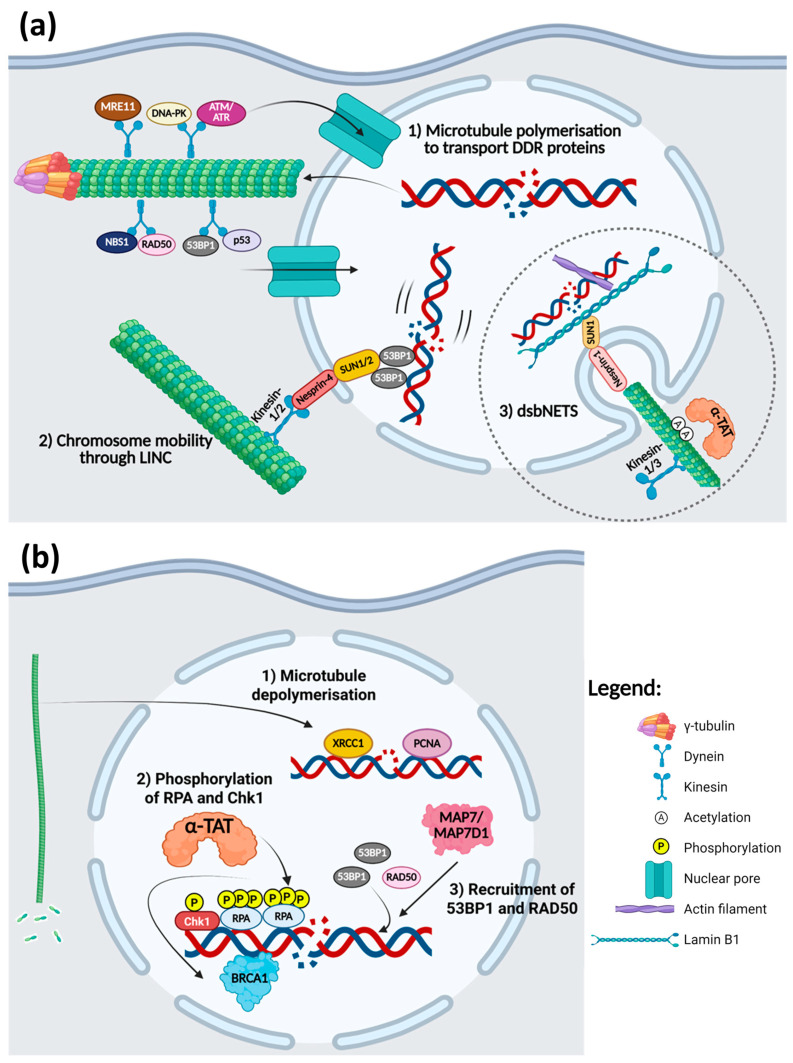
Microtubules and the DDR. (**a**) 1. γ-tubulin promotes the nucleation and polymerisation of microtubules in response to DNA damage that facilitates the nuclear translocation of several DDR proteins with the help of the dynein motor proteins. 2. Microtubules increase the movement of damaged DNA to the nuclear periphery by kinesin-1/2 linked to DSB-bound 53BP1 through the LINC complex. 3. α-TAT acetylates α-tubulin in the microtubules, which allows them to create invaginations on the nuclear membrane in tandem with kinesin-1/3, forming DSB-capturing nuclear envelope tubules (dsbNETs) with the help of LINC complex proteins, lamin B1, and actin filaments. *Created in BioRender. H, C.E. (2025)* https://BioRender.com/e81652n (accessed on 14 April 2025). (**b**) 1. Microtubule depolymerisation promotes the recruitment of XRCC1 and PCNA to SSBs in the DNA, thus augmenting BER. (2, 3) Function of other MAPs in DDR–2. α-TAT induces the hyperphosphorylation and phosphorylation of RPA and Chk1, respectively, which help the recruitment of BRCA1 to DSBs. 3. MAP7/MAP7D1 promotes the recruitment of 53BP1 and RAD50 to DSB sites. *Created in BioRender. H, C.E. (2025)* https://BioRender.com/lskqcz8 (accessed on 14 April 2025).

Correspondingly, microtubule dynamics could also modulate the DDR, as illustrated in Figure 2. DNA damage induction increases the accumulation of γ-tubulin and decreases the localisation of the DDR proteins in the centrosome [134]. Centrosomal γ-tubulin promotes the nucleation and polymerisation of the microtubules, which are responsible for the transport of DDR proteins, especially those involved in DSB repair, from the cytoplasm into the nucleus [134,136]. ATM, ATR, MRE11, Rad50, NBS1, DNA-PK, 53BP1, and p53 are transported by the dynein motor along microtubule bundles into the nucleus (Figure 2(a1)) [136]. Vincristine disruption of the microtubule network inhibits the translocation of these DDR proteins into the nucleus even when DNA damage is induced by doxorubicin [136]. γH2A.X foci are stabilised for a prolonged period when paclitaxel- or vincristine-treated NSCLC and breast cancer cells are irradiated, signifying that DDR and repair are impaired when microtubule dynamics are disrupted [136]. Additionally, disruption of the organisation of microtubules by mebendazole also results in the sequestration of NBS1 and Chk2 in the cytoplasm of glioma cells [137]. These DDR proteins are found exclusively in the nucleus even without DNA damage, but the inhibition of microtubule dynamics altered their localisation to the cytoplasm in the cells, which reduces the efficiency of DNA damage repair and decreases cell viability when these cells are irradiated [137].

Similar to their actin counterpart in HR repair, some studies have also shown that microtubule dynamics regulate the movement of chromatin after the induction of DNA damage [135,138,139]. Microtubules in the cytoplasm regulate chromatin mobility in the nucleus through the linker of nucleoskeleton and cytoskeleton (LINC) complex as presented in Figure 2(a2) [139]. Microtubule motor proteins, kinesin-1 and kinesin-2, interact with nesprin-4 spanning the outer nuclear membrane, which in turn interact with SUN1/2 located on the inner nuclear membrane to transmit the signals from the cytoplasm to the nucleus [139]. Altogether, DSB-induced microtubule dynamics and the LINC complex promote the mobility and movement of the DSBs through the 53BP1 proteins bound to DNA lesions [135,139]. This helps in the efficient repair of the DNA damage through NHEJ, as the movement of the DSBs augments the likelihood of the joining of broken DNA ends [135,139]. Disturbance to microtubule dynamics by microtubule stabilising or depolymerising drugs attenuated the movement of DSBs [139].

Shokrollahi et al. (2024) discovered that cytoplasmic microtubule dynamics, along with kinesin-1 and kinesin-3 (KIF5B and KIF13B), cause the nuclear envelope to invaginate inward to form a tubular network called DSB-capturing nuclear envelope tubules (dsbNETs), as shown in Figure 2(a3) [140]. The formation of dsbNETs is dependent on ATM/DNA-PKc signalling upon the DNA damage induction [140]. α-tubulin subunits are acetylated by α-tubulin acetyltransferase (α-TAT) on their lysine 40 residue when DSBs are induced, recruiting the kinesin motors and driving the invagination of the nuclear envelope to form dsbNETs through the LINC complex, nesprin-1 and SUN1, and the nuclear lamin B1 [140]. Altered expression of any of these proteins inhibits the formation of the dsbNETs. Actin filaments bound to the DSBs also help to stabilise the dsbNETs structure as well as promote the movement of DSBs, allowing them to be captured by the dsbNETS [140]. The repair centres formed at the dsbNETs, which are enriched with DDR proteins, promote the joining of the broken ends of the DNA, thus increasing the efficiency of DSB repair through NHEJ and HR [140]. This study shows how all three major components of the cytoskeleton are necessary for the formation and optimal function of the dsbNETs to direct DSB repair efficiently.

In addition, microtubule dynamics have a role in the modulation of the BER pathway, as displayed in Figure 2(b1). In cells treated with nocodazole, there is an increase in the accumulation of XRCC1 and PCNA at laser-induced DNA damage sites [114]. This suggests that microtubule depolymerisation can positively regulate BER in the event of DNA damage to counter the toxic effects of DNA damaging agents [114].

Upon the induction of DNA damage, MTBPs also have a role in regulating the DNA damage responses (Figure 2(b2,b3)). Apart from its role in the formation of dsbNETs, α-TAT also promotes the hyperphosphorylation of RPA and phosphorylation of Chk1 [141]. This fosters the recruitment of BRCA1 as well as activates the cell cycle checkpoint to induce S/G2 phase arrest [141]. Therefore, the knockdown of α-TAT resulted in decreased BRCA1 foci formation, a 50% reduction in the efficiency of HR repair, and reduced S/G2 phase arrest in HeLa and U2OS cells after treatment with camptothecin to induce DSBs [141]. However, it remains unknown whether these effects of the α-TAT knockdown are downstream of decreased α-tubulin acetylation or a direct effect on RPA and Chk1 acetylation, as other studies have shown that RPA activity could also be regulated through acetylation [141,142].

Microtubule-associated proteins MAP7 and MAP7D1, which help in the recruitment of kinesin-1 to the microtubules, have been found to interact with 53BP1, RAD50, BRCA1, MLH1, and XPC through their N-terminal regions [143]. Interestingly, these interactions were not affected by disruption to microtubule dynamics [143]. However, knockdown of MAP7D1 caused breast cancer cells to be arrested in the G1 phase, yet this effect is not as pronounced in the knockdown of MAP7 alone [143]. The double knockdown of MAP7 and MAP7D1 reduced the recruitment of 53BP1 to DSB sites and a decrease in the binding of RAD50 to chromatin after γ-irradiation, resulting in an increase in the number of DNA damage lesions after 8 h of irradiation [143].

### 4.3. Intermediate Filaments and DDR

**Figure 3:**
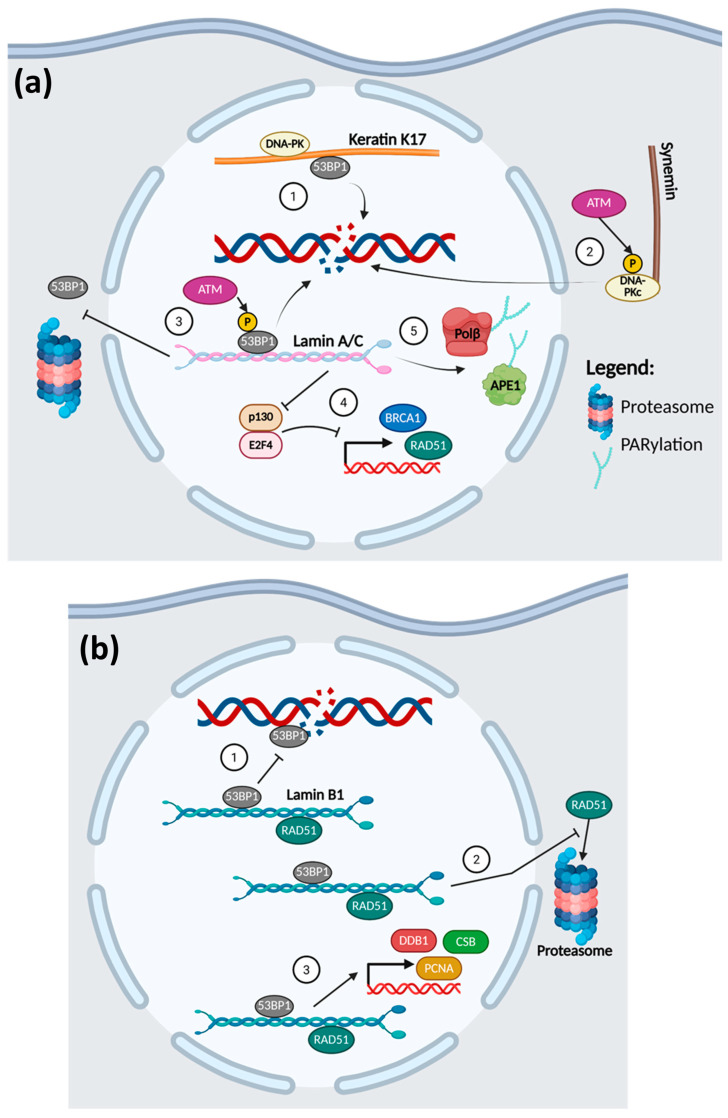
The roles of intermediate filaments in the DDR. (**a**) 1. Keratin K17 interacts with 53BP1 and DNA-PKc, promoting their recruitment to DSB sites. 2. Synemin acts as a scaffold for DNA-PKc, allowing its phosphorylation by ATM, which augments its recruitment to sites of DNA damage. (3–5) The roles of Lamin A/C in DDR–3. Lamin A/C helps in the retention of 53BP1 in the nucleus and prevents its proteasomal degradation. In the presence of DNA damage, ATM will phosphorylate 53BP1, causing it to dissociate from Lamin A/C to be recruited to DSB sites. 4. Lamin A/C prevents the formation of the p130/E2F4 repression complex, promoting increased expression of BRCA1 and RAD51 in the event of DNA damage. 5. Lamin A/C promotes the PARylation of APE1 and Polβ, increasing their activation and triggering BER. *Created in BioRender. H, C.E. (2025)* https://BioRender.com/tmikm41 (accessed on 14 April 2025). (**b**) Lamin B1 and the DDR. 1. The overexpression of lamin B1 sequesters 53BP1, preventing it from being recruited to DSB sites. 2. Lamin B1 promotes the retention of RAD51 in the nucleus upon DNA damage, preventing its proteasomal degradation. 3. Lamin B1 promotes the expression of NER proteins DDB1, CSB, and PCNA. *Created in BioRender. H, C.E. (2025)* https://BioRender.com/pobuek1 (accessed on 14 April 2025).

Since lamins are located solely in the nucleus, it is of no surprise that they take part in the DDR pathways. As illustrated in Figure 3, both A- and B-type lamins have been implicated in the repair pathways of DNA DSBs [144,145,146,147].

Both lamin types are associated with NHEJ repair of DSBs through their interaction with 53BP1. In the absence of DNA damage, 53BP1 is bound to lamins through its minimal focus region, which includes the Tudor and ubiquitin-dependent recruitment (UDR) domains [144,145]. Upon DSB induction, ATM phosphorylation of 53BP1 causes it to dissociate from the lamins to be recruited to the sites of DNA damage [144,145]. In human dermal fibroblast cells, the absence of A-type lamins (lamin A/C) prevents the retention of 53BP1 in the nucleus and causes it to undergo proteasomal degradation within the cytoplasm, thus making it unavailable to respond to DNA damage [144]. Although the knockdown of A-type lamins did not affect the stability of 53BP1 in osteosarcoma U2OS cells, it still caused a defect in the recruitment of 53BP1 to DSB sites [144]. Therefore, the loss of lamin A/C attenuates the formation of 53BP1 foci in DNA-damaged cells, impairing NHEJ repair and decreasing cell survival [144]. On the other hand, it is the overexpression of lamin B1 that prevents 53BP1 foci formation and efficient NHEJ repair [145]. Excess lamin B1 binds and sequesters 53BP1, preventing 53BP1 from being recruited to the sites of DNA damage [145].

Lamins are likewise involved in HR repair of DSBs. The expression levels of RAD51 and BRCA1 are dependent upon the expression of A-type lamin (lamin A/C) [146]. Its depletion in multiple cell types reduces the expression and impedes IR-induced RAD51 foci formation [146]. Redwood et al. (2011) reported that the loss of the A-type lamins causes p130 to complex with E2F4 to repress the expression of BRCA1 and RAD51, leading to deficient DNA HR repair and decreased cell survival after irradiation [146]. The B-type lamin, lamin B1, also interacts with RAD51 to maintain its stability by inhibiting its proteasomal degradation and promoting its nuclear localisation when DSBs are induced by ionising radiation [147]. This allows the formation of RAD51 repair foci in the nucleus, which increases the efficiency of HR repair and thus cell survival [147].

Some studies have also shown that A-type lamins have a role in the regulation of BER (Figure 3(a5)), while B-type lamin B1 is involved in NER (Figure 3(b3)) [148,149]. Lamin A/C regulates the PARylation of BER enzymes APE1 and Polβ, which is necessary for their optimal activities [148]. The depletion of lamin A/C decreases the post-transcriptional PARylation of APE1 and Polβ, reducing their activities and ability to respond to DNA damage [148]. Cells without lamin A/C are thus more susceptible to oxidative and alkylation damage of the DNA due to the defective BER response [148]. Meanwhile, silencing of lamin B1 causes the reduction of NER-related protein expression, namely damage-specific DNA binding protein 1 (DDB1), Cockayne syndrome protein B (CSB), and PCNA [149]. This results in a late initiation of NER in response to UV irradiation and increases apoptosis of the cells [149].

Nair et al. (2021) have shown that another intermediate filament, keratin K17, is involved in the DDR of DSBs [150]. Nuclear K17 was elucidated to interact with 53BP1, DNA-PKc, and γH2A.X, which are involved in the DDR choice pathway favouring NHEJ. In K17 knock-out epidermoid carcinoma cells, there was less DNA damage repair complex foci formation after X-ray radiation, as 53BP1 recruitment to the DSB sites was impaired (Figure 3a(1)). This led to increased cell death of the K17 knockout cells after DNA damage induction as compared to the K17-expressing cells [150].

As illustrated in Figure 3(a2), another IF that has been discovered to play a role in the DDR is synemin, a class IV filament [151,152]. The study by Deville et al. (2020) revealed that synemin knockdown in HNSCC cells reduces NHEJ activity and its overexpression increases it [152]. Synemin serves as a binding partner for DNA-PKc and helps in the modulation of its autophosphorylation [152]. With the downregulation of synemin, phosphorylation of DNA-PKc and its foci formation are lowered, resulting in a decrease in HNSCC cell survival upon DNA damage induction [152]. As synemin is frequently overexpressed in HNSCC, it could confer resistance to radiation therapy by upregulating NHEJ [152].

Class VI nestin may also be implicated in the DDR process. A study by Ma et al. (2014) elucidated that the knockdown of nestin in nasopharyngeal cancer cells induces spontaneous DNA damage and slows the DDR to irradiation-induced DNA damage [153].

**Figure 4:**
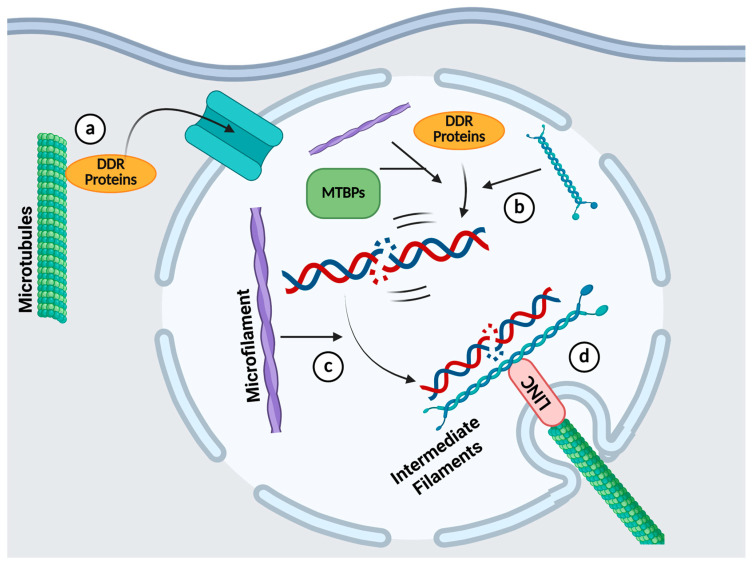
Overview of the cytoskeleton’s functions in DSB repair. (**a**) Microtubules aid in the translocation of DDR proteins into the nucleus. (**b**) Microtubules, microfilaments, and intermediate filaments help to recruit DDR proteins to DSBs. (**c**) Microfilaments regulate the movement of DSBs to the nuclear periphery. (**d**) Microtubules in tandem with lamin B1 form dsbNETs, promoting DSB repair. *Created in BioRender. H, C.E. (2025)* https://BioRender.com/axbarw0 (accessed on 14 April 2025).

Table 2 shows a summary of the roles that cytoskeletal molecules play in the regulation of different DDR pathways. Most of the studies involving the cytoskeleton and DDR were conducted by the induction of DSBs, and they distinctly exhibit the importance of all three components of the cytoskeleton in both NHEJ and HR repair processes (Figure 4). Microtubules are required in the translocation of key DSB repair proteins from the cytoplasm into the nucleus upon DNA damage induction, and their recruitment to the DSBs is dependent upon cytoskeletal molecules from the three classes. Moreover, actin and its binding proteins are needed to promote the movement of DSBs to the nuclear periphery, where microtubules form dsbNETs with the help of lamin B1, which helps in the clustering of the damaged DNA. This increases the efficiency of the DSB repair. Hence, this suggests that a novel way to inhibit the DDR in cancer therapy is to target the cytoskeleton in tandem. However, the function of the cytoskeleton in other DDR pathways has not been extensively researched. As discussed above, a couple of investigations have shown that cytoskeletal molecules are also involved in these DDR processes, such as in BER and NER, but the mechanisms have not been clearly elucidated. The roles of the cytoskeleton in MMR and TLS have also not been reported. Deeper investigations into the interplay between the DDR and cytoskeleton are needed to develop novel therapies that leverage this synergistic relationship.

## 5. Targeting the Cytoskeleton in Conjunction with DNA Damage Induction in Cancer

Some of the most commonly used forms of cancer therapy include both radiotherapy and chemotherapy, which rely on the induction of DNA damage in the cancer cells directly or indirectly to trigger its apoptosis [7]. Radiotherapy is used in more than 50% of cancer treatment worldwide, and chemotherapy is prevalently utilised in the treatment of late-stage disease [7,154]. Although they have proven to be useful in the treatment of many cancer types, resistance impedes their overall effectiveness [155]. Radiotherapy treatment typically involves the introduction of either DSBs or SSBs due to the induction of reactive oxygen species (ROS) during irradiation [7,156]. This will trigger the activation of the corresponding DDR pathways. Successful repair of the induced DNA damage will allow the tumour cell to survive and re-enter the cell cycle, thus contributing to radioresistance [155]. Chemotherapeutics, e.g., alkylating agents, antimetabolites, platinum-based compounds, antitumour antibodies, and topoisomerase inhibitors, also introduce DNA damage directly or by affecting the cell replication process [7]. Depending on the type of chemotherapeutic drugs, there are many mechanisms that the tumour cells could employ to resist chemotherapy-induced cell death [157]. Similar to radioresistance, the utilisation of DDR pathways to repair DNA damage and evade the targeting contributes significantly to chemoresistance [158].

Interest in the use of specific DDR pathway inhibitors has emerged as evidence from in vitro discoveries shows that these agents can be used to sensitise cancer cells to radiation or chemotherapy. By inhibiting DNA repair in tumour cells, they would cause an accumulation of DNA damage within the cell, leading to cell death [159]. Four PARP inhibitors, Olaparib, Rucaparib, Niraparib, and Talazoparib, have been approved by the FDA for treatment of BRCA-dependent breast, ovarian, pancreatic, and prostate cancers [160,161]. Their use in combination with other drugs such as bevacizumab, enzalutamide, and abiraterone in these cancers are currently assessed in phase III clinical trials [160]. Other DDR inhibitors targeting DNA-PK, ATM, ATR, Chk1, Wee1, and Pol θ are likewise being investigated in multiple Phase I/II clinical trials as monotherapeutic agents or in combination with other agents [160]. This corresponds to results from earlier in vivo studies, which have shown that combination therapies of specific DDR inhibitors with radiotherapy or chemotherapy could result in a more efficient killing of tumour cells than standalone radio- or chemotherapy treatments [155]. For example, Chk1 synergised with cisplatin to induce cell death of small cell lung cancer [162].

Although DDR inhibitors are a promising line of agents to be used in conjunction with DNA damaging treatments in cancer, their use has been limited due to the toxicities observed in healthy tissues [160]. More improvements have to be done to widen their therapeutic range [163]. Additionally, since different types of DNA damage are repaired by specific DDR pathways, it is difficult to inhibit all of them to fully sensitise the cancer cells to radio- or chemotherapy, which inflicts diverse DNA damage types [157]. Therefore, other ways of inhibiting the DDR pathways more broadly in cancer therapies should be explored to obtain a more efficacious treatment regime.

Since the cytoskeleton is intimately involved in the DDR process, the combination of DNA damaging treatment, DDR inhibitors, and cytoskeletal-targeting drugs may have a more universal effect on preventing DNA repair and increasing the efficacy of cancer treatments. This is exhibited by the combination of carboplatin, a DNA alkylating agent, and paclitaxel, a microtubule-targeting agent (MTA), which are widely used in the treatment of cancers such as ovarian, cervical, and NSCLC with PARP inhibitors. Although the addition of paclitaxel increased carboplatin-induced DNA damage in the cancer cells, combining veliparib, a PARP inhibitor, to this regime promoted the progression-free survival in patients with BRCA+ breast cancer [164,165]. A phase I study also observed that the combination of ceralasertib, an ATR inhibitor, with paclitaxel is more well-tolerated than with carboplatin [166]. More importantly, this combination demonstrates durable efficacy against chemotherapy-resistant melanoma [166].

Other studies have likewise suggested a potential in the combination of MTAs and DDR inhibitors for cancer treatment. Docetaxel, a taxane, is used as a first-line therapy in several malignancies, including advanced prostate cancer [167]. However, resistance frequently occurs, limiting further treatment options [168,169]. A study by Chao and Goodman (2021) shows that when prostate cancer cells develop resistance to docetaxel, they are more resistant to DNA damage induced by the MTA due to alteration in the expression of DDR genes [170]. Of these genes, DNA-PKc has been found to be significantly upregulated, and treating docetaxel-resistant prostate cancer cells with DNA-PKc specific inhibitors, such as NU7441, LTURM3, and M3814 (which are under clinical investigation), resensitised the cells to docetaxel treatment [170]. DNA-PKc-specific inhibitors also helped to overcome the cross-resistance of docetaxel-resistant prostate cancer cells to cabazitaxel, a taxane used in second-line therapy for prostate cancer [170]. In a study by Zhang et al. (2023), comprehensive dose-response screening of DDR inhibitors with other anti-cancer therapeutics showed that various MTAs in combination with either ATM, ATR, or DNA-PK inhibitors have the greatest efficacy across 62 different cancer cell lines [171]. These studies suggest that further investigation of combining DDR inhibitors with anti-cytoskeletal agents may yield more efficacious novel therapies.

However, apart from MTAs such as vinca alkaloids, taxanes, and epothilones, not many cytoskeleton-specific drugs have been approved for clinical use in cancer therapies [172]. Presently, there are no FDA-approved anti-actin filament-specific agents, and of the many types of intermediate filaments, only vimentin inhibitors are available [172]. The reasons why cytoskeletal-targeting drugs are so limited despite the cytoskeleton being shown to play an intricate role in the development and progression of cancer are wide-ranging. For MTAs, resistance to the agents is one of the biggest hurdles in developing one effective for use in cancer therapy [173,174]. Another obstacle to overcome in the development of cytoskeletal targeting drugs is the off-target cardiac, hepatic, and renal toxicities that they often exhibit in vivo, since the cytoskeleton is also involved in normal physiological function [172,173].

Furthermore, adopting a precision-based approach in the development of anti-cytoskeleton drugs for cancer treatment is likely necessary to ensure that promising cytoskeleton-disrupting agents will be targeted specifically to the cancer cells to minimise their off-target effects [173]. There have been some advances in this for MTAs with the use of antibody-drug conjugates (ADCs) [175,176]. The antibody directs its drug conjugates specifically to the tumour cells, and the whole ADC complex will be taken up by the cell, whereafter the drug could exhibit its cytotoxic effects [176]. A number of these ADCs have been approved, with more under clinical trials, for clinical use in various cancer types [173,176]. Therefore, determining their effectiveness in combination with a DNA damage-based cancer therapy is worth looking into as a potentially more efficacious treatment option. Another potential approach is the development of photoswitchable analogues of common MTAs, such as paclitaxel, epothilone, and combretastatin A4, whose activation is dependent on light [177]. However, most of these compounds are still in the early stages of development, and currently none of them could be used to target solid tumours in the body [177]. Metabolic instability and maintaining high potency with reversible photoswitchability also pose challenges for the adoptability of these compounds in clinical use [177]. Nevertheless, this is a promising area of research that could provide specificity to MTAs, which would allow them to be more widely used in combination with other treatment modalities such as DDR inhibitors.

There are many small molecules targeting actin and its ABPs available, which were developed as cancer therapeutics [172,178]. However, none are used clinically for cancer treatment due to their off-target effects and general toxicities [172,178]. Therefore, novel anti-actin or ABP agents are needed to fulfil this gap to be able to disrupt actin dynamics to inhibit cancer. Applying similar strategies as MTAs to develop antibody-drug conjugates for precisely targeted anti-microfilament agents is a promising area to be explored. Following this, their inhibition of DDR pathways for the treatment of various cancers could be studied. Although determining an appropriate monoclonal antibody (that has a specific antigen on the tumour cell), linker, and drug compound combination is a considerable task, the ability to do so will fulfil an unmet gap in the ability to target actin filaments specifically for cancer treatment [176]. For example, using these techniques, nuclear actin filaments could be disrupted to prevent DDR protein recruitment to DSB sites and the clustering of DSBs in the nuclear periphery.

Other possible ways of harnessing the role of the cytoskeleton in the DDR include the inhibition of the binding of cytoskeletal components to the DNA or DDR proteins. For example, the interaction between nuclear myosin I/V or the INO80 complex and the damaged DNA could be targeted to prevent the movement and clustering of DSBs. γH2A.X signalling that induces Arp2/3 to initiate nuclear actin filament formation is another promising area that could be targeted to reduce HR efficiency. Another possibility is to prevent dynein binding to key DSB repair proteins to prevent DDR proteins from being transported into the nucleus. However, more in-depth mechanistic studies to foster a deeper understanding of the interaction between the cytoskeletal molecules and DDR proteins or DNA are needed to guide the design of drugs targeting the respective protein candidates. The targeting of the cytoskeleton to inhibit DDR is thus a greatly unexplored area that has a lot of potential to advance novel and more efficacious cancer therapies.

## 6. Conclusions

All three major classes of the cytoskeleton have been shown to play significant roles in the various pathways of the DNA damage response processes. They are involved in the expression of some DDR proteins (intermediate filaments), their nuclear translocation (microtubules), their recruitment to sites of damage in the DNA, and the movement and clustering of damaged DNA (microfilaments) to enriched repair sites in the nuclear periphery. This provides opportunities for disruption of the cytoskeleton in cancer treatments dependent on inflicting DNA damage on tumour cells. However, not many compounds are available clinically to target these cytoskeletal molecules. Hence, this presents an unmet need which necessitates novel drug development strategies based on incorporating these potentially beneficial therapeutic targets in combination regimens with existing DDR inhibitors, chemotherapy, or radiotherapy. The function of the cytoskeleton in the DDR is a major area that is not widely explored in the research field. With more interest in this field, breakthroughs could be made by more deeply understanding the role of the cytoskeleton in DDR in cancer, which may contribute to disease progression by inducing resistance in cancer treatment. Furthermore, it could promote the invention of novel technologies and techniques to develop more specific efficacious therapies.

## Figures and Tables

**Table 2:** Summary of the cytoskeleton’s roles in the DDR.

Cytoskeletal Class	Key Proteins	DDR Pathway	Impact on the DDR	Ref.
Microfilaments	Nuclear actin, Formin-2 and Spire-1/2	NHEJ	Nuclear actin polymerisation by Formin-2 and Spire-1/2: - Increases NHEJ by retention of Ku80 on DSBs. - Promotes BER through the accumulation of XRCC1 on sites of DNA damage.	[110,113,114]
		BER		
	Filamin-A, Arp2/3, Nuclear actin, Myosin I and V, INO80 complex	HR	- Filamin-A regulates BRCA2 recruitment to DSBs. - Actin filament and its motor proteins regulate damaged DNA movement to the nuclear periphery.	[115,116,117,118,119,122,123,124,128]
	Cytoplasmic actin, JMY, Myosin IIa	p53	Myosin IIa increases p53 retention and accumulation in the nucleus, while JMY acts as its transcription co-activator, augmenting its target gene expression.	[109,111,112,129]
	INO80 complex	NER	INO80 complex helps to recruit XPA and XPC to the DNA to initiate NER.	[125]
Microtubules	αβ-Tubulin, γ-TubulinDynein	NHEJ and HR	Microtubule dynamics and dynein help in the transport of DSB repair proteins, such as ATM, MRN11, NBS1, etc., into the nucleus upon DNA damage induction.	[134,136,137]
	αβ-Tubulin, Kinesin-1/2, MAP7/MAP7D1	NHEJ	- MAP7/MAP7D1 helps in the recruitment of 53BP1 and RAD50 to DSB sites. - Microtubule dynamics promote the movement of DSBs through the LINC complex attachment to 53BP1, which increases their end joining.	[135,138,139,143]
	α-TAT, αβ-Tubulin, Kinesin-1/3	NHEJ and HR	Promote the creation of dsbNETs with the help of the LINC complex, lamin B1, and actin filament.	[140]
	α-TAT	HR	Promotes hyperphosphorylation of RPA and phosphorylation of Chk1, increasing BRCA1 recruitment to DSB sites.	[141,143]
	αβ-Tubulin	BER	Microtubule depolymerisation increases XRCC1 and PCNA accumulation on damaged DNA for BER to take place.	[114]
Intermediate Filaments	Lamin A/C, Synemin, K17	NHEJ	- Lamin A/C fosters the nuclear retention of 53BP1 and its recruitment to DSBs. - Synemin promotes the activation of DNA-PKc. - K17 aids 53BP1 and DNA-PKc recruitment to DSB sites.	[144,150,152]
	Lamin B1	NHEJ	Inhibits NHEJ as overexpression sequesters 53BP1 from being recruited to DSBs.	[145]
	Lamin A/C, Lamin B1	HR	- Lamin A/C increases BRCA1 and RAD51 expression by inhibiting formation of p130/E2F4 repression complex. - Lamin B1 increases the stability and nuclear accumulation of RAD51.	[146,147]
	Lamin A/C	BER	Fosters the PARylation of APE1 and Polβ to increase their activities.	[148]
	Lamin B1	NER	Expression of NER key proteins, DDB1, CSB, and PCNA, is dependent upon the presence of lamin B1.	[149]

## Abbreviations

The following abbreviations are used in this manuscript:

α-TAT	α-tubulin acetyltranferase
ABP	Actin binding protein
ADC	Antibody-drug conjugate
AML	Acute myeloid leukemia
ATM	Ataxia Telangiectasia Mutated
BER	Base excision repair
CSC	Cancer stem cells
CTL	Cytotoxic T-cells
DDR	DNA damage response
DSB	Double stranded break
dsbNETs	DSB-capturing nuclear envelope tubules
ECM	Extracellular matrix
EMT	Epithelial-mesenchymal transition
HNPCC	Hereditary non-polyposis colorectal cancer
HNSCC	Head and neck squamous cell carcinoma
HR	Homologous recombination
ICL	Interstrand cross-links
IF	Intermediate filaments
JMY	Junction-mediating and regulatory protein
LINC	Linker of nucleoskeleton and cytoskeleton
MAP	Microtubule-associated proteins
MET	Mesenchymal-epithelial transition
MMR	Mismatch repair
MRN	MRE11/RAD50/NBS1 complex
MTA	Microtubule targeting agent
MTBP	Microtubules binding protein
MTOC	Microtubule organising centre
NER	Nucleotide excision repair
NHEJ	Non-homologous end joining
NK cell	Natural killer cell
NSCLC	Non-small cell lung cancer
OGG1	Oxoguanine glycosylase 1
PCNA	Proliferating cell nuclear antigen
PTM	Post-translational modification
ROS	Reactive oxygen species
RPA	Replication protein A
SSB	Single strand break
TLS	Translesion synthesis
